# Evaluation of the Effects of Increasing Standard Uncertainty on the Combined Uncertainties: Case of an IE2 5.5 kW Induction Motor

**DOI:** 10.3390/s26072161

**Published:** 2026-03-31

**Authors:** Edoardo Fiorucci, Andrea Fioravanti, Simone Mari, Giovanni Bucci, Fabrizio Ciancetta, Alberto Prudenzi

**Affiliations:** Department of Industrial and Information Engineering and Economics, University of L’Aquila, 67100 L’Aquila, Italy; andrea.fioravanti@univaq.it (A.F.); simone.mari@univaq.it (S.M.); giovanni.bucci@univaq.it (G.B.); fabrizio.ciancetta@univaq.it (F.C.); alberto.prudenzi@univaq.it (A.P.)

**Keywords:** induction motor efficiency, measurement uncertainty, efficiency measurement, induction motor testing, loss separation method, energy efficiency, electrical machine metrology, electric motor losses

## Abstract

Developing electric motors with higher efficiencies for energy savings and environmental protection is crucial. The efficiency of grid-connected induction motors can be measured using various approaches; the preferred method is the indirect approach, which evaluates the separate losses from the additional losses due to residual losses. This approach follows the traditional approach to efficiency determination, introducing experimental procedures to assess additional losses by measuring the torque delivered by the motors. As noted in previous articles, the procedure is complex and requires numerous direct measurements. One area of interest is the determination of measurement uncertainty. This work aims to quantify the sensitivity of the combined uncertainties of losses and efficiency to variations in directly measured input variables: power frequency, rotational speed, torque, power, current, voltage, resistance, coolant temperature, and cold frame temperature. The results presented here help select measurement instrumentation, depending on whether the tests are aimed solely at determining efficiency or whether it is necessary to analyze the trend of the various types of loss, as occurs in optimization and experimental verification processes with high-performance materials, based on a comprehensive analysis of all standard and combined uncertainties, and with experimental data to assign a realistic value to the uncertainties themselves.

## 1. Introduction

Induction motors, widely used in industrial, commercial, and domestic applications, account for over 85% of all rotating electrical machines. In industrialized countries, these motors consume over 60% of the electrical energy [1]; even small increases in efficiency can generate significant energy savings on a large scale [2,3]. For this reason, IEC/EN 60034-30-1 [4], published in March 2014, harmonized energy efficiency classes for electric motors, defining four International Efficiency (IE) classes for mains-operated AC motors. Measurement techniques have also evolved. To ensure the correct assessment of the efficiency levels defined by IEC/EN 60034-30-1 [4], IEC/EN 60034-2-1 [5] defines suitable low-uncertainty test methods (the preferred methods in [5]). Efficiency values can only be compared and verified if they are based on the same test method. Furthermore, the manufacturer must document the type of test performed to determine the efficiency value [5].

Since the efficiency of induction motors is very high (over 96% for high power motors), one of the most critical aspects in the current normative is mainly related to the determination of the related efficiency measurement uncertainty.

The JCGM 100, Guide to the expression of Uncertainty in Measurement (GUM) [6] establishes general rules for evaluating and expressing uncertainty in measurement; its application is mandatory in all sectors. When reporting the result of a measurement of a physical quantity, it is obligatory that some quantitative indication of the quality of the result be given so that those who use it can assess its reliability. Without such an indication, measurement results cannot be compared, either among themselves or with reference values given in a specification or standard. Regarding the measurement uncertainty of efficiency (or other quantities), the ISO/IEC 17025 standard [7], “General requirements for the competence of testing and calibration laboratories”, is the international reference for laboratories worldwide that carry out calibration and testing activities. The 2017 revision of [7] introduced a requirement that laboratories must take measurement uncertainty into account when reporting results against a specification or requirement. This requires identifying and assessing the uncertainty components for each method. Using the indirect method is very complex, as shown in several works in the literature [8,9]. The indirect method is unsuitable for high-efficiency motors, where the uncertainty in measuring losses, given their small values, produces high relative uncertainties and possible energy IE classification errors. Since the 2-1-1B method [5] is preferred by several companies [10,11] for testing low- and medium-power three-phase induction motors, it is important to analyze the uncertainties it entails [12].

Several studies have addressed the challenges of efficiency estimation in electric drives. Paramo-Balsa et al. presented a non-invasive method for estimating the load and efficiency of induction motors without interrupting their operation, minimizing estimation errors [13]. Machado and Flesch proposed a structured method for differential efficiency measurement for drive systems and for quantifying measurement uncertainty [14]. Leighton and Akasapu applied an uncertainty quantification framework consistent with GUM standards to efficiency tests of electric drive units, propagating measurement chain uncertainties to the derived efficiency value [15]. Similarly, Hölsch and Wallscheid developed a measurement model to evaluate power and efficiency uncertainty across the entire torque-speed plane of test benches for three-phase drives [16], while Tiihonen et al. studied efficiency measurements of converter-fed induction motors according to IEC 60034-2-3 [17]. Overall, all authors agree that small increases in efficiency require low-uncertainty, expensive, and impractical measurement instruments. Conversely, this work demonstrates that, due to the non-uniform nature of uncertainty propagation, not all measured quantities impact the final result equally: some measurements are more sensitive and therefore require higher-performance instrumentation, while others can be reliably acquired with less expensive sensors.

The aim of this work is to investigate which measures are critical, regarding uncertainty, in the evaluation of the efficiency of a motor, to direct the efforts in the right direction.

The remainder of this paper is organized as follows. Section 2 presents the methodology and the fundamental equations underlying the mathematical treatment adopted in this study. Section 3 describes the experimental setup used for testing the induction motor. Section 4 reports the test results. Finally, Section 5 discusses the results with particular emphasis on the adequacy and implications of the evaluated measurement uncertainties.

## 2. Methods

The procedure for measuring efficiency using the 2-1-1B method presented in [5] is rather complex, since this method requires the determination of individual loss components: (i) stator windings losses [18], (ii) rotor windings losses [19], (iii) iron losses [20,21], (iv) friction and windage losses, and (v) additional losses. The evaluation of these losses [22,23,24,25] requires the repeated measurement of several quantities: (i) cold motor shell temperature, θ_0_; (ii) stator winding resistance, R_0_; (iii) rotor winding resistance if the motor has a wound rotor. The test procedure consists of the following main operational phases: (i) measurement of the winding resistance; (ii) test at rated load; (iii) test at variable load; (iv) no-load test.

The total losses P_T_ are then calculated [5,26]:

(1) $$P_{T}=P_{s,\theta }+P_{r,\theta }+P_{fw}+P_{fe}+P_{LL}\;$$

where P_s,θ_ are the corrected stator losses, P_r,θ_ are the corrected rotor losses, P_fw_ are the friction and windage losses, P_fe_ are the iron losses and P_LL_ are the additional losses. The nominal (at the nominal load) efficiency is:

(2) $$\eta =\frac{P_{1,\theta }-P_{T}}{P_{1,\theta }}$$

where P_1,θ_ is the absorbed power corrected at the temperature determined by the nominal load test. The efficiency is therefore equal to:

(3) $$\eta =1-\frac{P_{s,\theta }+P_{r,\theta }+P_{fw}+P_{fe}+P_{LL}}{P_{1,\theta }}$$

Since the motor’s efficiency depends on the load percentage, this calculation must be repeated for different load percentages. From Equation (4), it is possible to calculate the uncertainty in determining the efficiency u_c_(η) [5,6]:

(4) $$u_{c}\left(\eta \right)=\sqrt{\begin{array}{c} {\left(\frac{P_{s,\theta }+P_{r,\theta }+P_{fw}+P_{fe}+P_{LL}}{P_{1\theta }^{2}}\right)}^{2}\cdot {u\left(P_{1\theta }\right)}^{2}+\\ +{\left(-\frac{1}{P_{1\theta }}\right)}^{2}\cdot \left({u\left(P_{s,\theta }\right)}^{2}+{u(P_{r,\theta })}^{2}+{u\left(P_{fw}\right)}^{2}+{u(P_{fe})}^{2}+{u\left(P_{LL}\right)}^{2}\right) \end{array}}$$

where u(P_s,θ_), u(P_r,θ_), u(P_fe_), u(P_fw_), u(P_LL_), u(P_1,θ_) are the combined uncertainties related to the losses P_s,θ_, P_r,θ_, P_fw_, P_fe_, P_LL_, P_1θ_ measured at the rated load.

To calculate the uncertainty u_c_(P_s,θ_) it is necessary to sequentially calculate Equations (5)–(7) [5,6].

(5) $$u_{c}\left(\theta_{w}\right)=\sqrt{{\left(\frac{\theta_{0}+235}{R_{0}}\right)}^{2}\cdot u^{2}\left(R_{w}\right)+{\left(\frac{R_{w}*\left(\theta_{0}+235\right)}{{R_{0}}^{2}}\right)}^{2}\cdot u^{2}\left(R_{0}\right)+{\left(\frac{R_{w}}{R_{0}}\right)}^{2}\cdot u^{2}\left(\theta_{0}\right)}$$

(6) $$u_{c}\left(k_{\theta }\right)=\sqrt{{\left(\frac{1}{235+\theta_{w}}-\frac{235+\theta_{w}+25-\theta_{c}}{{\left(235+\theta_{w}\right)}^{2}}\right)}^{2}\cdot {u^{2}}_{c}\left(\theta_{w}\right)+{\left(-\frac{1}{235+\theta_{w}}\right)}^{2}\cdot u^{2}\left(\theta_{c}\right)}$$

(7) $$u_{c}\left(P_{s,\theta }\right)=\sqrt{{\left(3I\cdot R_{N}\cdot k_{\theta }\right)}^{2}\cdot u^{2}\left(I\right)+{\left(1.5I^{2}\cdot k_{\theta }\right)}^{2}\cdot u^{2}\left(R_{N}\right)+{\left(1.5I^{2}\cdot R_{N}\right)}^{2}\cdot u^{2}\left(k_{\theta }\right)}$$

**Figure 1:**
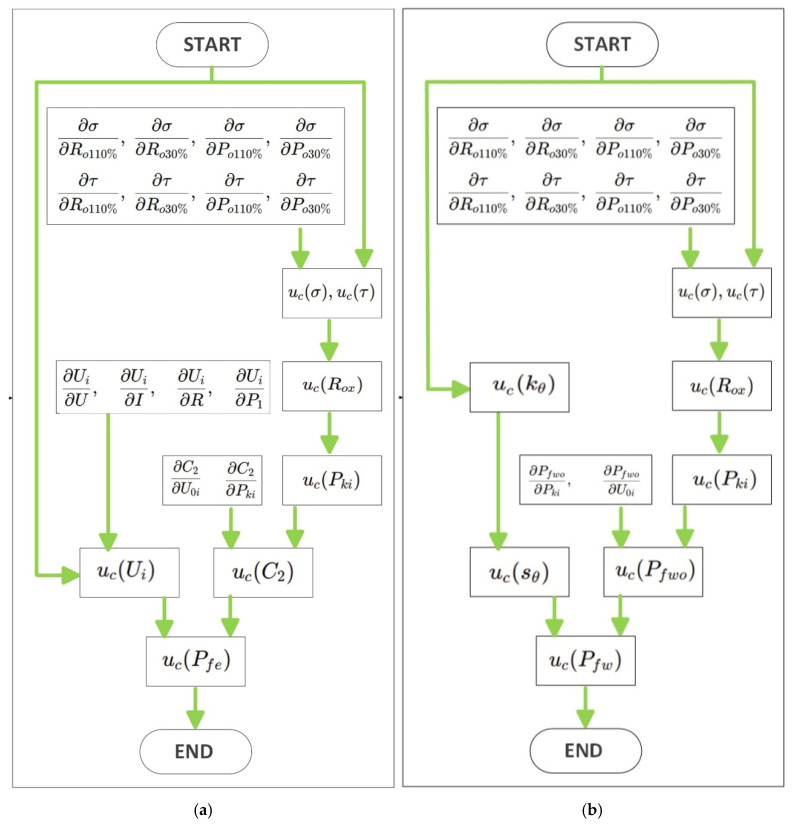
Flowchart of the uncertainty propagation procedure used to evaluate the combined standard uncertainty of: (**a**) iron losses $u_{c}(P_{fe})$, (**b**) friction and windage losses $u_{c}(P_{fw})$.

**Figure 2:**
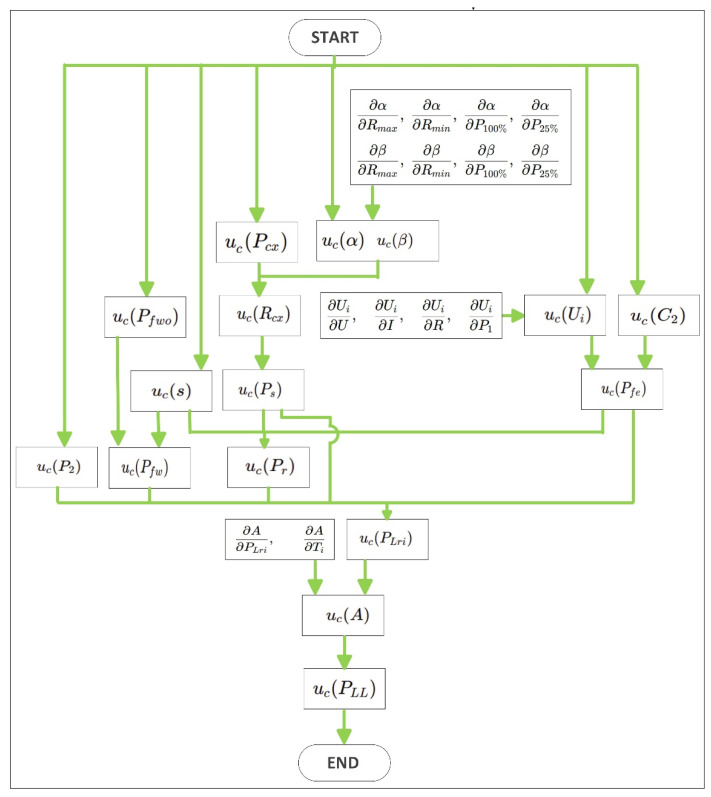
Flowchart of the uncertainty propagation procedure used to evaluate the combined standard uncertainty of u_c_(P_LL_).

The calculation of the combined uncertainties associated with the separate loss measurements requires complex steps, which for brevity are outlined in the flow charts in Figure 1, the iron u_c_(P_fe_), friction and ventilation u_c_(P_fw_), and Figure 2 for additional losses u_c_(P_LL_), with the temperature corrections required by [5].

The losses in the rotor winding for the nominal load test are equal to:

(8) $$P_{r}=(P_{1}-P_{s}-P_{fe})\cdot s$$

where s is the slip.

If the cooling fluid has a temperature other than the standard value of 25 °C, it is necessary to introduce a temperature correction using the nominal load slip referred to as the reference temperature of the cooling fluid $s_{\theta }$:

(9) $$P_{r,\theta }=(P_{1}-P_{s,\theta }-P_{fe})\cdot s_{\theta }$$

According to the general expression of the combined uncertainty, the uncertainty u_c_(P_r,θ_) in the determination of P_r,θ_, is:

(10) $$u_{c}\left(P_{r,\theta }\right)=\sqrt{{\left(s_{\theta }\right)}^{2}\cdot \left(u^{2}\left(P_{1}\right)+u_{c}^{2}\left(P_{s,\theta }\right)+u_{c}^{2}\left(P_{fe}\right)\right)+{\left(P_{1}-P_{s,\theta }-P_{fe}\right)}^{2}\cdot u_{c}^{2}\left(s_{\theta }\right)}$$

When the cooling fluid is not at 25 °C, the active power absorbed in the test at nominal load must also be corrected according to the expression:

(11) $$P_{1,\theta }=P_{1}-(P_{s}-P_{s,\theta }{+P}_{r}-P_{r,\theta })$$

from which we have:

(12) $$u_{c}\left(P_{1,\theta }\right)=\sqrt{u^{2}(P_{1})+u_{c}^{2}\left(P_{s}\right)+u_{c}^{2}\left(P_{s,\theta }\right)+u_{c}^{2}\left(P_{r}\right)+u_{c}^{2}\left(P_{r,\theta }\right)}$$

To calculate u_c_(P_1,θ_), it is necessary to evaluate the uncertainties u_c_(P_s,θ_) and u_c_(P_r,θ_), which are not corrected for temperature and are related to the nominal load test.

The iron losses under rated-load conditions are evaluated as:

(13) $$P_{fe}=C_{2}\cdot U_{i}^{2}$$

According to the general expression of combined standard uncertainty, the uncertainty associated $u_{c}(P_{fe})$ can be expressed as:

(14) $$u_{c}(P_{fe})=\sqrt{U_{i}^{4}\;u_{c}^{2}(C_{2})+{\left(2\;C_{2}\;U_{i}\right)}^{2}\;u_{c}^{2}(U_{i})}$$

where $C_{2}$ is the slope of the straight line obtained from the relationship between $P_{fe0}$ and $U_{0}^{2}$, while $U_{i}$ is the internal voltage.

Since the values of $U_{i}$ and $C_{2}$ are known, the evaluation of $u_{c}(P_{fe})$ requires the preliminary determination of the uncertainties $u_{c}(C_{2})$ and $u_{c}(U_{i})$. Therefore, the uncertainty analysis must be carried out for both quantities before propagating them to the final expression of the iron-loss uncertainty. Figure 1a summarizes the uncertainty propagation procedure adopted for the evaluation of the combined standard uncertainty $u_{c}(P_{fe})$. Starting from the directly measured quantities, the uncertainties of the linear model parameters σ and τ (slope and intercept), used to determine $R_{0x}$, are first evaluated. These contributions are then propagated through the intermediate quantities $R_{0x}$, $P_{ki}$, $C_{2}$, and $U_{i}$, up to the final uncertainty associated with $P_{fe}$.

The friction and windage losses corrected to rated-load conditions are evaluated as:

(15) $$P_{fw}=P_{fw0}\;(1-s_{\theta }{)}^{2.5}$$

According to the general law of propagation of uncertainty, the combined standard uncertainty $u_{c}(P_{fw})$ is given by:

(16) $$u_{c}(P_{fw})=\sqrt{\left(1-s_{\theta }{)}^{5}\;u_{c}^{2}(P_{fw0})+{\left[2.5\;P_{fw0}\;(1-s_{\theta }{)}^{1.5}\right]}^{2}u_{c}^{2}(s_{\theta }\right)}$$

where $P_{fw0}$ denotes the friction and windage losses at no load, and $s_{\theta }$ is the rated-load slip referred to the reference cooling-fluid temperature.

Since $s_{\theta }$ and $P_{fw0}$ are known, the evaluation of $u_{c}(P_{fw})$ requires the preliminary calculation of the uncertainties $u_{c}(s_{\theta })$ and $u_{c}(P_{fw0})$. Therefore, these two contributions must be determined before propagating them to the final uncertainty of the corrected friction and windage losses. Figure 1a shows the flowchart adopted to calculate the combined standard uncertainty $u_{c}(P_{fw})$ by propagating the uncertainties of the directly measured quantities through the intermediate variables $R_{0x}$, $P_{ki}$, $P_{fw0}$, $k_{\theta }$, and $s_{\theta }$.

The additional load losses under rated-load conditions are evaluated as:

(17) $$P_{LL}(T)=A\cdot T^{2}$$

According to the general law of propagation of uncertainty, the combined standard uncertainty associated $u_{c}(P_{LL})$ can be expressed as:

(18) $$u_{c}(P_{LL})=\sqrt{T^{4}\;u_{c}^{2}(A)+{\left(2\;A\;T\right)}^{2}u^{2}(T)}$$

where A is the coefficient of the quadratic relationship between the additional load losses and the torque, and T is the torque under rated-load conditions.

Since the values of T, u(T), and A are known, the evaluation of the uncertainty associated with the additional load losses requires the determination of the uncertainty $u_{c}(A)$. Therefore, the uncertainty analysis must first be carried out for this coefficient before propagating it to the final expression of $u_{c}(P_{LL})$.

Figure 2 shows the uncertainty propagation path used for the determination of the combined standard uncertainty of $u_{c}(P_{LL})$. The procedure starts from the directly measured quantities and propagates their uncertainty through the intermediate quantities used to calculate $P_{2}$, $P_{s}$, $P_{r}$, $P_{fe}$, and $P_{fw}$. These terms are then combined to evaluate the uncertainty of the residual losses $u_{c}(P_{Lri})$, from which the uncertainty of the coefficient A is obtained. Since A is the slope of the linear relationship between the additional load losses and the square of the torque, its uncertainty is finally propagated to determine $u_{c}\left(P_{LL}\right).$

## 3. Test Setup

The previously described method was applied to process data acquired during the experimental characterization of the induction motor under test. The main technical specifications and rated operating parameters of the machine are reported in Table 1

**Figure 3:**
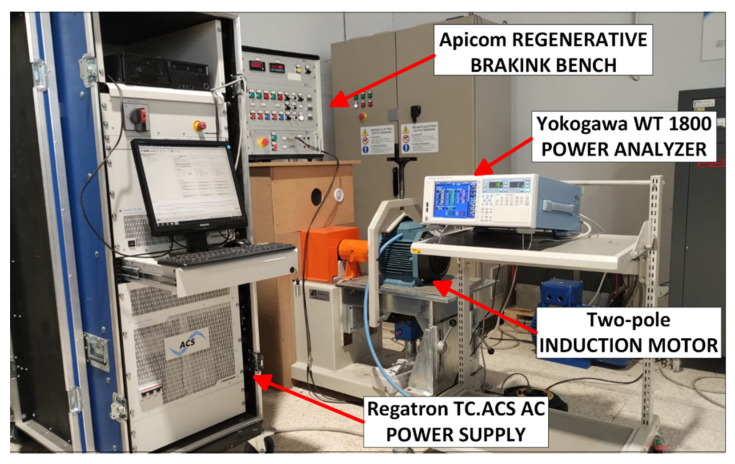
Experimental setup of electric motor test bench and data acquisition system.

The tests were carried out with an APICOM regenerative braking bench (Apicom Cento, Italy) with a nominal power of 21 kW, equipped with a 100–200 Nm dual scale torque transducer, with a precision class of 0.1% and rotational speed measurement with an encoder of 1024 pulses per revolution, with an uncertainty of ± 1 rpm. The system was supplied by a REGATRON TC.ACS regenerative AC power supply (Regatron AG, Rorschach, Switzerland) operating as a programmable grid simulator, providing a voltage total harmonic distortion of 0%. Voltage, current, and power were measured with a Yokogawa WT 1800 power analyzer (Yokogawa Electric Corporation, Tokyo, Japan), with direct insertion for line current measurements. Winding resistances were measured with a Chauvin Arnoux CA 6255 micro-ohmmeter (Chauvin Arnoux, Asnières-Sur-Seine, France). We used a Fluke 971 thermo-hygrometer (Fluke, Everett, WA, USA) to measure the cooling air temperature and a Fluke 561 m (Fluke, Everett, WA, USA) for the motor casing temperature, see Figure 3.

The directly measured quantities are:

- supply frequency;
- rotational speed;
- torque;
- input power;
- current;
- voltage;
- stator winding resistance;
- cooling fluid temperatures;
- cold motor casing temperature.

It is necessary to obtain 96 quantities, each with its respective combined uncertainty, as outputs to obtain the values of the separate losses and the efficiency. The total amount of data is 340, including all inputs and outputs. To simplify the processing of measured parameters, a software application has been developed in the NI LabVIEW environment to determine the efficiency of induction motors in accordance with IEC 60034-2-1 [11]. In more detail, the program calculates separate and total losses and provides the standard uncertainties of all indirect quantities as outputs. The application is extensively illustrated in [10], which describes the subVIs implemented for each step of the calculation process.

The uncertainties with which the various quantities are measured are all set to the conventional value of 0.1% except one, for which a variability of between 0.1% and 10% is imposed, to highlight the effect of this variation on the final uncertainty.

In this way, it is possible to directly assess the sensitivity of the resulting uncertainty to the measurement uncertainty of each individual quantity. This will allow us to understand which measurements are most critical and which actions are necessary to improve performance in terms of uncertainty.

## 4. Results

The analysis, according to the procedure for the indirect efficiency based on the segregation of losses, starts from the winding resistances measured at ambient temperature and proceeds through the rated-load test, from which stator and rotor winding losses are determined. Then, the load curve test and the no-load test are used to evaluate the constant losses (iron losses, friction and windage losses). Finally, the additional load losses is estimated and, consequently, the motor efficiency.

The results obtained from the experimental tests are first summarized in Table 2, which condenses the main information related to the performed measurements, the calculated results, and the associated uncertainty.

Once the values of the related quantities had been determined, the standard uncertainties of all directly measured quantities were initially set equal to 0.1%. Starting from this baseline configuration, the standard uncertainty of a single directly measured quantity was varied while keeping the others fixed to evaluate its effect on the combined uncertainties of the separate losses, total losses, and efficiency.

The data obtained were then organized into two series of graphs. The first series represents the sensitivity of the combined uncertainties of the separate and total losses and of the efficiency to the variation in the standard uncertainty of one of the directly measured quantities; the data help evaluate which measurement uncertainties should be controlled since they have a greater effect on the combined uncertainties, and, consequently, which instrumentation should be chosen with better performance and which can instead be of lower performance without this having an appreciable effect.

The second data set compares the effects, on a single combined uncertainty, of varying the standard uncertainties of all directly measured quantities. The graphs can be used to identify the directly measurable quantities whose uncertainty must be controlled to reduce the uncertainty of the quantity under consideration.

### 4.1. Impact of Standard Uncertainty on Losses and Efficiency

**Figure 4:**
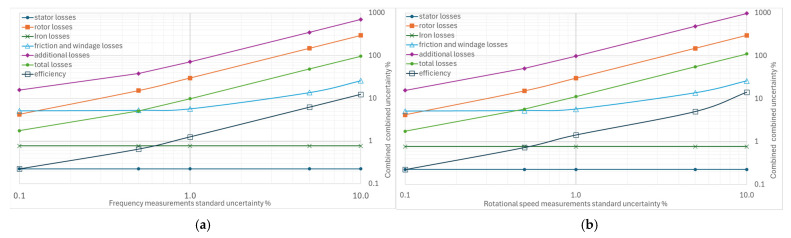
Effects of increasing standard uncertainty on separate and total losses, and efficiency: (**a**) of the supply frequency, (**b**) of rotational speed.

**Figure 5:**
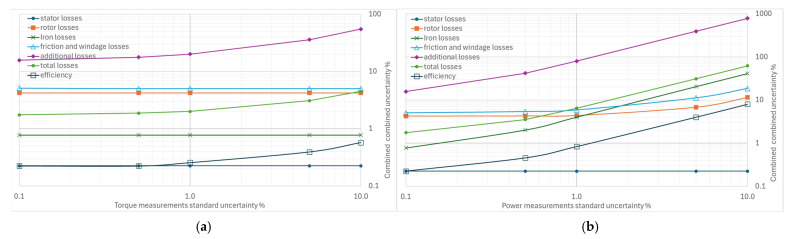
Effects of increasing standard uncertainty on separate and total losses, and efficiency: (**a**) of torque, (**b**) of input power.

**Figure 6:**
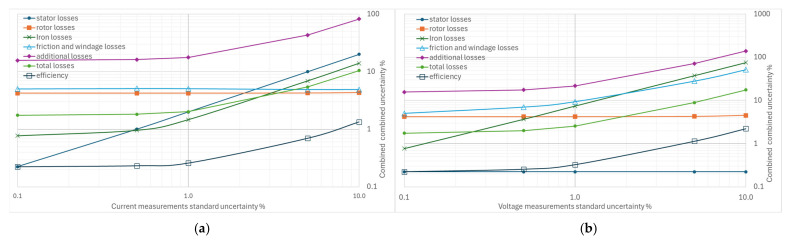
Effects of increasing standard uncertainty on separate and total losses, and efficiency: (**a**) of stator current, (**b**) of input voltage.

**Figure 7:**
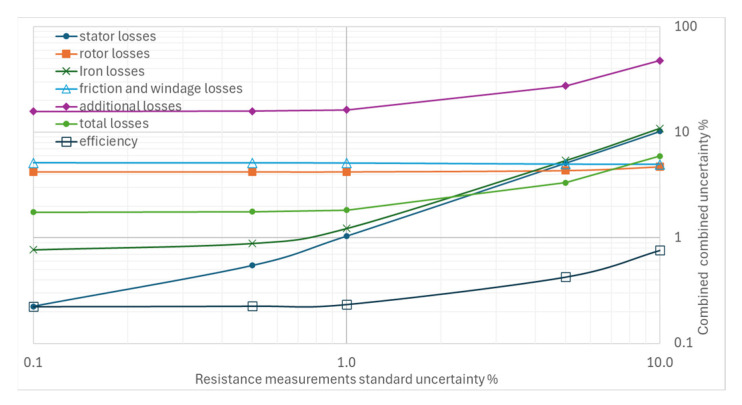
Effects of increasing standard uncertainty on separate and total losses, and efficiency of resistance measurements.

**Figure 8:**
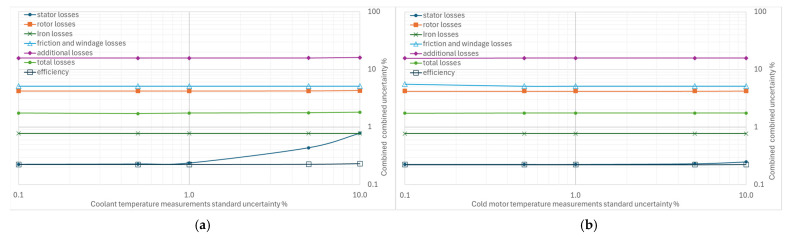
Effects of increasing standard uncertainty on separate and total losses, and efficiency: (**a**) of the cooling fluid, (**b**) of cold motor shell temperature.

From Figure 4, Figure 5, Figure 6, Figure 7 and Figure 8, the percentage variation in the combined uncertainties relative to the measurement uncertainty of the quantity under examination is shown on a logarithmic scale.

Figure 4a illustrates the effect of increasing the standard uncertainty of the supply frequency from 0.1% to 10% on the measurement of the separate losses, total losses, and efficiency. The combined uncertainties most affected by the supply frequency uncertainty are the additional losses and the rotor losses: variations of 0.1% to 10% in the standard uncertainty of frequency produce a combined uncertainty range of 15.7% to 702.9% for additional losses and of 0.22% to 12.4% for rotor losses. These increases are also reflected in total losses and efficiency, as shown by their curves. The effect is negligible for friction and ventilation losses, without appreciable impact on the other types of losses.

Figure 4b refers to the effect of increasing the standard uncertainty of the rotational speed. Given the similarity between rotational speed and frequency (strictly connected through slip in the induction motor), the same considerations that have been made can already be applied.

The standard uncertainty of the torque measurement has a minor effect on the combined uncertainties, as shown in Figure 5a, where only a slight increase is observed, except for the combined uncertainty of the additional losses. In fact, they are determined with a regression method based on torque measurements. This result is not unexpected: the processing of separate losses with additional losses derived from residual losses is an evolution of the technique used in previous editions of the same standard, in which the additional losses were conventionally hypothesized to 0.5% of the machine’s rated power.

From the graph in Figure 5b, the effect of increasing standard uncertainty on the combined efficiency uncertainty is moderate, with values ranging from 0.22% to 0.83% for variations in input power uncertainty from 0.1% to 1%. The uncertainty in iron losses is more sensitive than that in rotor losses, friction, and ventilation losses. At the same time, there is no effect on stator losses where input power measurements are not used in the calculation.

The stator current measurement’s standard uncertainty (Figure 6a) has a limited effect on all the losses considered and on efficiency. The combined uncertainty of efficiency remains below 1% even for current uncertainty values up to 5%. Stator losses are most affected, with significant increases, while the effects on iron-loss uncertainty are negligible. No changes are recorded for rotor losses and for friction and ventilation losses. The additional losses’ uncertainty is sensitive to the uncertainty of the current measurement but much less than the uncertainty of the power; for example, if an uncertainty of 1% is considered in both power and current measurements, the combined uncertainties of the additional losses are 79% to 17%, respectively.

The effect of variations in the uncertainty of the voltage measurements in Figure 6b is slightly more significant than that of the current on the efficiency uncertainty; this is a consequence of the greater sensitivity to these variations shown by the combined uncertainties of both the iron losses and those due to friction and ventilation, while the effect of the additional losses on the combined uncertainty is still evident and more significant.

The standard uncertainty of resistance measurements has a limited effect on the combined efficiency uncertainty; in the range of 0.1% to 10%, the efficiency uncertainty varies from 0.22% to 0.76% (Figure 7). There is no effect on the combined rotor uncertainties and on friction and ventilation losses. The impact on additional losses is less than in the above-mentioned cases.

The variations in the uncertainties of the cooling fluid (Figure 8a) and cold motor shell temperature (Figure 8b) measurements have virtually no effect on the combined uncertainties, except for the stator losses, which are affected by the uncertainty of the coolant temperature measurement.

### 4.2. Impact of Increasing Individual Uncertainties on Combined Uncertainty

**Figure 9:**
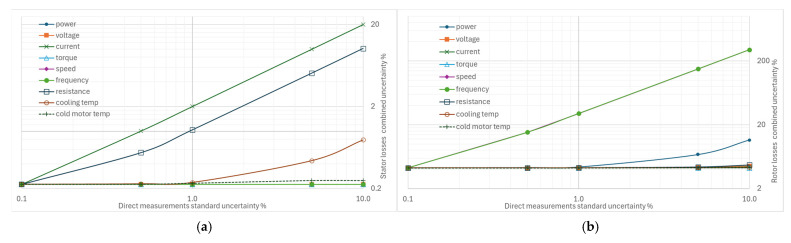
Effects on the combined uncertainty due to increasing other standard uncertainties: (**a**) for stator winding losses, (**b**) for rotor winding losses.

Figure 9a shows the effects on the combined uncertainty for the measurement of stator winding losses, P_s,θ_. In line with the definition of stator losses, the combined uncertainty is sensitive to the uncertainty in the measurement of stator current and, to a lesser extent, to that in the measurement of winding resistance. Although the losses are corrected to the coolant temperature, increasing the uncertainty of this measurement has a marginal effect. The uncertainties of the other quantities do not influence this type of uncertainty.

The graph in Figure 9b (effects on the combined uncertainty for the measurement of rotor winding losses) confirms that the rotation speed and frequency measurements must be performed with appropriate uncertainties to limit the combined uncertainty in the rotor loss measurement. On the contrary, variations in the other uncertainties have no significant effects, except for that of the electric power, in a marginal form.

**Figure 10:**
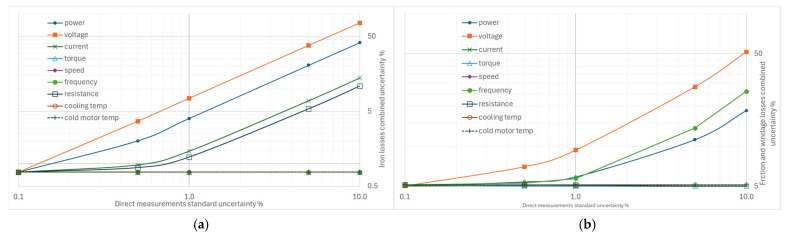
Effects on the combined uncertainty due to increasing other standard uncertainties: (**a**) for iron losses, (**b**) for friction and windage losses.

The behavior of the combined uncertainty of the iron losses measurement in Figure 10a indicates that the most crucial standard uncertainties to monitor are in the voltage and power measurements; the effects of uncertainty in both stator current and winding resistance measurements are not negligible.

The standard uncertainty to which the composite uncertainty of friction and windage losses is most sensitive is that of the voltage measurement, followed by that of the supply frequency measurement, as illustrated in Figure 10b. The uncertainty in the measured absorbed power is also relevant.

**Figure 11:**
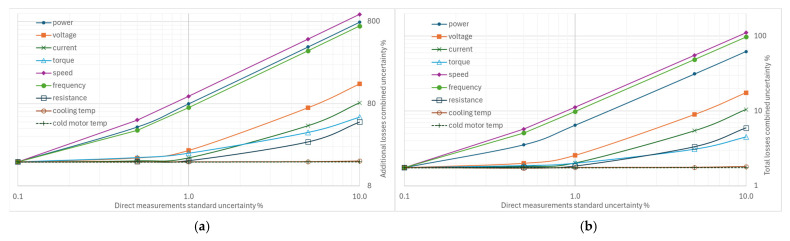
Effects on the combined uncertainty due to increasing other standard uncertainties: (**a**) for additional losses, (**b**) for total losses.

From Figure 11a, it appears that variations of 0.1% to 10% of the uncertainties in the direct measurements can lead to excessively high values, up to 500% or 900% of the combined uncertainty in additional losses. The standard uncertainties with the most marked effects concern the measurement of rotational speed, electrical power, and supply frequency. On the contrary, the measurements of current and voltage, torque, and winding resistance have a smaller effect.

Analysis of the combined uncertainty data for total losses shows that variations in the standard uncertainty of rotational speed measurement are the most significant, even more so than those in electrical power measurement, followed by frequency measurement. The effects of uncertainties in voltage and current measurements are much smaller, as shown in Figure 11b.

**Figure 12:**
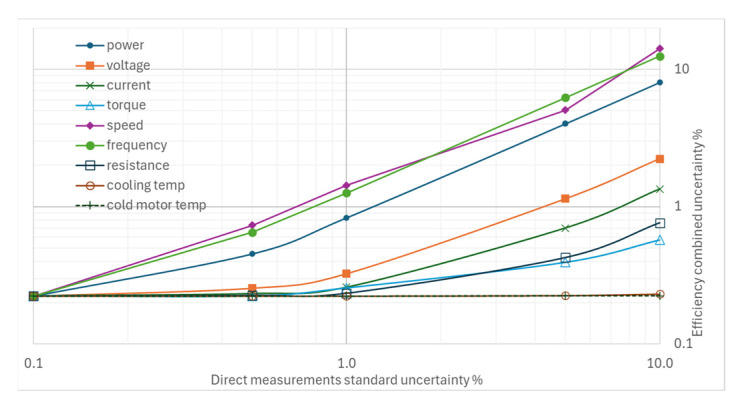
Effects on the combined uncertainty due to increasing other standard uncertainties for efficiency.

The most significant results in Figure 12 relate to the sensitivity of the combined efficiency uncertainty to the standard uncertainties of rotational speed and power frequency measurements; their effects are greater than those of electrical power and voltage measurements, a result that was inconceivable prior to the elaborations presented in this paper. The variation in torque measurement uncertainty, however, is insignificant, and variations in temperature measurement uncertainties have no appreciable effect.

## 5. Discussions

The uncertainty analysis of the supply frequency emphasizes the importance of selecting instrumentation with an appropriate full-scale range. When accuracy is specified as a percentage of full scale, the absolute uncertainty remains constant across the measurement range; therefore, the relative uncertainty increases significantly at low readings. To minimize this effect, the instrument’s full-scale value should be chosen as close as possible to the maximum expected measurement. Instead, in IEC 60034-2-1, the supply frequency measurement accuracy is specified only as ±0.1% of full scale, implying improved relative accuracy near the upper range limit and degraded performance at lower values. The results also show that the determination of additional losses is the dominant contributor to efficiency uncertainty. These losses are obtained by subtraction; evaluating a small quantity as the difference between two large measured values amplifies uncertainty even when individual percentage uncertainties are small. While uncertainty in additional losses has limited relevance during motor design optimization, accurate rotor loss measurement is critical for experimental validation of efficiency-oriented models and high-efficiency performance assessment.

Considering the uncertainty associated with rotational speed, torque and speed transducers commonly have full-scale ranges of 6000 rpm or higher; therefore, for us, uncertainties of the order of ±0.5% or higher are possible for machines with multiple pole pairs, for which rated rotational speeds are lower than 1500 or 1000 rpm. Instead, IEC 60034-2-1 specifies that rotational speed measurements should be accurate within 0.1 rpm.

Based on the uncertainty results associated with torque transducers, it can be observed that the use of high-performance torque sensors is not strictly necessary. This finding is economically significant, as commercial transducers with 0.1% accuracy can cost between €5000 and €10,000 each, and multiple units may be required when testing motors of different sizes.

For electrical measurements of input power, IEC 60034-2-1 specifies instruments with an accuracy class of 0.2 for direct tests and 0.5 for indirect methods. As highlighted by the present results, these instruments must ensure an overall uncertainty of 0.2% of the reading at a power factor of 1.0, including the effects of transducers when used. Indeed, the influence of additional losses on the combined uncertainty is critical; even small increases in input power measurement uncertainty can lead to substantial increases in overall uncertainty.

An important outcome related to the standard uncertainty of stator current measurements is that instruments with higher uncertainty may be acceptable when measurements are intended solely for efficiency evaluation during motor testing. However, during the design and optimization phase, when stator losses must be experimentally validated, high-performance ammeters are recommended. This differentiated approach can lead to appreciable cost savings.

The effect of variations in voltage measurement uncertainty suggests that, when the objective is limited to efficiency certification, instruments with less stringent voltage accuracy may be acceptable. Conversely, high-performance voltmeters are required when the focus is on evaluating iron losses and validating the selection of appropriate magnetic materials.

The standard uncertainty associated with resistance measurements indicates that the use of high-performance ohmmeters is not critical for efficiency evaluation. However, such instruments are necessary for the accurate experimental determination of stator and iron losses.

Variations in the uncertainties associated with the cooling fluid temperature and the cold motor shell temperature indicate that low-performance instrumentation can be used for both measurements without affecting overall measurement quality.

Furthermore, the results concerning total losses partially contradict the traditional approach to motor testing, in which uncertainty in angular velocity measurement is often considered less significant than uncertainties in electrical quantities. Similarly, the uncertainty associated with supply frequency is frequently neglected, since tests are typically performed with the motor powered directly from the mains and the frequency is therefore assumed to be known and essentially constant.

## 6. Conclusions

The efficiency of mains-powered induction motors can be determined using different methods. The most widely adopted recommended method is based on determining separate losses and quantifying additional losses from the residual losses. The standard indicates reference values for the uncertainties of the measurements of the input quantities. Still, there are no targets for uncertainties during loss processing or objectives for the quality of the output efficiency value.

In this work, the effects of increased uncertainties in input quantities have been evaluated to quantify, using data from a real case of a motor tested in the laboratory, the order of magnitude of the sensitivity of the combined uncertainties in losses and efficiency. The most interesting results concern the criticality of uncertainties in the measurement of supply frequency and speed, whose variations have effects even more critical than those of the uncertainty in input power, whereas the variations in the uncertainties of torque and resistance measurements are less so. The effects of the variations in uncertainty in temperature measurements are not relevant. The uncertainties in voltage and current measurements significantly affect some types of loss, but do not affect the overall efficiency to a great extent. The aim of providing initial indications of the performance requirements for the measurement instrumentation appears to have been achieved. In future work, we intend to extend the analysis to a set of engines of different sizes to confirm the trends observed in this case study.

## Figures and Tables

**Table 1:** Rated parameters of the tested induction motor.

Parameter	Value
Motor type	Three-phase induction motor
Number of pole pairs	1
Rated power	5.5 kW
Rated voltage	380–420 V Δ
Rated current	10.7 A
Rated frequency	50 Hz
Rated speed	2900 rpm
Rated power factor	0.88
Efficiency class	IE2

**Table 2:** Summary of the experimental tests and associated uncertainties for the tested motor.

Quantity	Value	Uncertainty
Cold motor shell temp θ_0_ [°C]	17.8	±0.6
Cold motor winding res. R_0_ [Ω]	1.3530	±0.0006
Hot motor winding R_w_ [Ω]	1.7163	±0.0007
Coolant temp. θ_c_ [°C]	24.1	±0.3
Rated load Voltage rms U_n_ [V]	229.7	±0.065
Rated load Current rms I [A]	10.32	±0.004
Rated load Power P_1_ [W]	6209	±5.47
Rated load Rotational speed n [rpm]	2903	±0.6
Rated load Torque T [Nm]	18.20	±0.06
Rated load Frequency F [Hz]	50.009	±0.014
No load Current rms I_o_ [A]	4.296	±0.001
No load Voltage rms U_o_ [V]	229.3	±0.065
No load Power [W]	227	±1.57
Corrected input power (P_1_,_θ_)	6242.681	±41.032
Stator losses P_sθ_ [W]	274.955	±0.366
Rotor winding losses P_re_ [W]	188.547	±40.646
Iron losses P_fe_ [W]	119.013	±3.632
Friction and windage losses (P_fwo_) [W]	6242.681	±41.032
Residual losses P_Lr_ [W]	26.8391	±41.2107
Additional losses P_LL_ [W]	55.537	±28.2818
Total losses (P_t_) [W]	705.405	±49.667
Indirect efficiency (η)	0.887	±0.008

## Data Availability

The data are available from the corresponding author upon reasonable request.

## Nomenclature

A	Slope of the linear relationship between the additional load losses and $T^{2}$
$C_{2}$	Slope of the straight line relating $P_{fe0}$ to $U_{0}^{2}$
*F*	Supply frequency
I	Stator current
$I_{0}$	No-load current
$k_{\theta }$	Temperature correction factor
n	Rotational speed
$P_{1}$	Active power absorbed during the rated-load test
$P_{1,\theta }$	Active power corrected to the reference temperature
$P_{2}$	Output mechanical power
$P_{fe}$	Iron losses
$P_{fe0}$	Iron losses at no load
$P_{fw}$	Friction and windage losses corrected to rated-load conditions
$P_{fw0}$	No load friction and windage losses at
$P_{k}$	Constant losses independent of the load
$P_{LL}$	Additional load losses
$P_{Lr}$	Residual losses
$P_{r}$	Rotor winding losses
$P_{r,\theta }$	Rotor winding losses corrected to the reference temperature
$P_{s}$	Stator winding losses
$P_{s,\theta }$	Stator winding losses corrected to the reference temperature
$P_{T}$	Total losses
$R_{0}$	Stator winding resistance at no load test
$R_{N}$	Stator winding resistance at rated condition
$R_{w}$	Stator winding resistance measured after the test
s	Induction motor slip
$s_{\theta }$	Rated-load slip referred to the reference cooling-fluid temperature
T	Torque
$U_{0}$	No-load supply voltage
$U_{n}$	Rated-load voltage
$u_{c}(x)$	Combined standard uncertainty
$\theta_{0}$	Cold motor shell temperature
$\theta_{c}$	Cooling-fluid temperature
$\theta_{w}$	Stator winding temperature after the test
α	slope of the straight line of the stator resistance
β	intercept of the straight line of the stator resistance
σ	slope of the linear relationship of stator resistance under no-load test condition
τ	Intercept of the linear relationship of stator resistance under no-load test condition
η	Efficiency
